# Cellulose/Grape-Seed-Extract Composite Films with High Transparency and Ultraviolet Shielding Performance Fabricated from Old Cotton Textiles

**DOI:** 10.3390/polym15061451

**Published:** 2023-03-14

**Authors:** Xiaoqian Ji, Zhen Xu, Xinqun Xia, Zhaoning Wei, Jun Zhang, Guangmei Xia, Xingxiang Ji

**Affiliations:** 1 Key Laboratory of Pulp and Paper Science & Technology of Ministry of Education, State Key Laboratory of Biobased Material and Green Papermaking, Faculty of Light Industry, Qilu University of Technology (Shandong Academy of Sciences), Jinan 250353, China; 2 Beijing National Laboratory for Molecular Sciences, CAS Key Laboratory of Engineering Plastics, Institute of Chemistry, Chinese Academy of Sciences (CAS), Beijing 100190, China

**Keywords:** cellulose films, grape seed extracts, transparency, anti-ultraviolet, packaging field

## Abstract

Plastics displaying many merits have been indispensable in daily life and they still maintain the strong momentum of development. Nevertheless, petroleum-based plastics possess a stable polymer structure and most of them are incinerated or accumulated in the environment, leading to devastating impacts on our ecology system. Thus, exploiting renewable and biodegradable materials to substitute or replace these traditional petroleum-derived plastics is an urgent and important task. In this work, renewable and biodegradable all-biomass cellulose/grape-seed-extract (GSEs) composite films with high transparency and anti-ultraviolet performance were fabricated successfully from pretreated old cotton textiles (P-OCTs) using a relatively simple, green, yet cost-effective, approach. It is proved that the obtained cellulose/GSEs composite films exhibit good ultraviolet shielding performance without sacrificing their transparency, and their UV-A and UV-B blocking values can reach as high as nearly 100%, indicating the good UV-blocking performance of GSEs. Meanwhile, the cellulose/GSEs film show higher thermal stability and water vapor transmission rate (WVTR) than most common plastics. Moreover, the mechanical property of the cellulose/GSEs film can be adjusted by the addition of a plasticizer. Briefly, the transparent all-biomass cellulose/grape-seed-extracts composite films with high anti-ultraviolet capacity were manufactured successfully and they can be used as potential materials in the packaging field.

## 1. Introduction

It is well-known that plastics, with their combination of many merits, have been indispensable in our daily life since they were firstly developed [1,2,3]. Meanwhile, with the rapid development of technology and the increasing demands of people’s living standard, traditional plastics still maintain a strong momentum of development [2,4,5]. It was reported that the total mass of virgin plastics reached 8300 million metric tons by 2017 and approximately 370 million tons of plastics were manufactured in 2019 alone [3,6]. However, petroleum-based plastics possess a stable polymer structure and most of these petroleum-based plastics are incinerated or accumulated in the environment, leading to devastating impacts to our ecology [3,7,8]. Unfortunately, plastic waste debris has been found in most ocean basins, posing huge potential threats to animals and humans [9,10,11,12]. In addition, plastic recovery may form part of the solution, but it is often costly, time-consuming and energy intensive. Therefore, it is urgent to exploit renewable and biodegradable materials to substitute or replace these petroleum-derived plastics [13,14,15,16].

Biomass resources, including the three categories of animals, plants and microbes resources, are ubiquitous in our planet [17,18,19,20]. Biomass-based materials, such as collagen-based materials [21,22], starch-based materials [23,24], chitosan-based materials [25,26], chitin-based materials [27,28], cellulose-based materials [29,30] and so on, derived from these biomass resources have been developed rapidly and display wide applications in various fields, such as textiles and agriculture, pharmaceutical/biomedicine, food packaging, energy and water treatment. As the most widely distributed and abundant polysaccharide in nature, cellulose has attracted more and more attention recently and the commercialization of viscose fibers, cellophane (regenerated cellulose film) and celluloids (cellulose nitrate) have been recognized as significant alternatives to non-degradable plastics [7,29,30,31,32]. Nevertheless, these cellulose-based products are mostly produced from high-grade pulps (wood pulps or cotton liners) [33,34,35]. Besides their high cost, the manufacturing processes are usually tedious, harsh and harmful to the environment [7,34,36]. Furthermore, most of these cellulose-based products lack anti-ultraviolet capacity when they are exposed in outdoors. By contrast, developing more feasible and green methods to prepare anti-ultraviolet cellulose-based materials by employing less expensive cellulose sources seems to be more attractive and meaningful.

In fact, many works have been conducted to fabricate biomass-based products from low-cost agricultural and forestry residues [37,38,39]. Hu et al. reported a cost-effective way to prepare a new lignocellulosic bioplastic which is biodegradable, recyclable, scalable and strong from bagasse, grass and wheat straw [3]. Similarly, high-added-value biomass/polypropylene (PP) composites were successfully manufactured by coupling agricultural and forestry wastes with the petroleum-based plastics [40]. Meanwhile, biorefineries and biomass-residue valorization have become more and more popular recently, with the energy shortage and environmental pollution [41,42]. In addition, solid wastes from households, such as waste newspaper [34], paper cups [43], old textiles [44] and corrugated papers [45], are also attractive alternatives to the high-cost raw materials. It was found that waste newspapers can be transformed into regenerated cellulose films and aerogels to be used as packaging and adsorption materials [34,46,47]. Meanwhile, waste paper cups have been successfully converted into cellulose-based film with or without peeling off the polyethylene (PE) coating and paper–plastic composites with certain hydrophobicity were fabricated successfully [48]. Moreover, cellulose-based films with some anti-ultraviolet capacity can be also obtained by adopting corrugated paper as a source [45]. Additionally, waste polyester–cotton blended textiles were also completely valorized using the ionic liquid solvent method to achieve polyethylene terephthalate (PET) film and transparent regenerated cellulose films [44]. Therefore, the valorization of low-cost raw materials by fabricating cellulose-based materials with high value does bring benefits to the economy, ecology and society, and more attempts need to be made in the future.

In our previous work, anti-ultraviolet cellulose-based films including cellulose/aramid nanofibers (ANFs) and cellulose poly (meta-phenylene isophthalamide) (PMIA) were prepared successfully [35,49]. Nevertheless, aramid are synthetic fibers and cellulose/ANFs or cellulose/PMIA films are not completely biodegraded. Furthermore, the preparing process involved some toxic solvents, such as dimethyl acetamide (DMAc). Herein, renewable and biodegradable all-biomass cellulose/GSEs composite films with high transparency and anti-ultraviolet performance are fabricated from the waste cotton textiles and grape-seed extracts (GSEs) using a relatively simple, green, yet cost-effective, approach. To the best of our knowledge, most of the previous works related to GSEs are focused on their antioxidant capacity [50,51,52,53]. In contrast to the previous investigations, the structure and anti-ultraviolet performance of the cellulose/GSEs composite films were studied systematically in this work.

## 2. Materials and Methods

### 2.1. Chemicals and Materials

Old cotton textiles (OCTs), which have been worn for at least 7 years, were collected from household and used after pretreatment referred to our previous work [35]. Grape seed extracts (GSEs, food grade, ≥95.0%) were bought from the Diao Pharmacist Nutrition Restaurants (Dalian, China) and used as received. Deionized water was self-produced. The room-temperature ionic liquid, 1-allyl-3-methylimidazolium chloride (AmimCl), was used without any purification after donation by ICCAS-Henglian Biobased Materials Co., Ltd. (Weifang, China) [54].

### 2.2. Fabrication of Cellulose/GSEs Composite Materials

**Figure 1:**
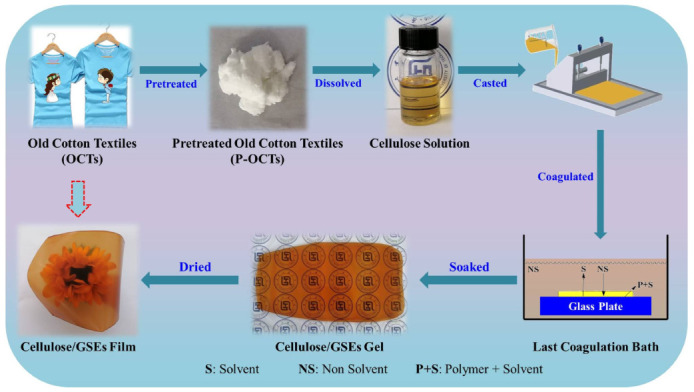
Schematic illustration of preparing process of cellulose/GSEs composite films. Adapted from Ref. [48].

Figure 1 exhibits the preparing process of cellulose/GSEs composite films. Firstly, the collected old cotton textiles (OCTs) were bleached with household disinfectant and washed thoroughly. Then, the pretreated old cotton textiles (P-OCTs) were dried and shredded by the household wall breaker for further use. Subsequently, 4.8 g of the crushed old cotton textiles (OCTs) were put into the 155.2 g of AmimCl and they were stirred vigorously for 180 min at around 80 °C to obtain the dissolved cellulose. Subsequently, the solution was cast on the glass plate to obtain liquid film with 0.1 cm in thickness and the plate was immersed into the deionized water coagulation to obtain the cellulose hydrogels. Lastly, the cellulose hydrogels were put into the GSEs/glycerol aqueous solution to achieve the cellulose/GSEs composite hydrogels, where the mass content of GSEs was 0%, 0.25%, 0.5%, 1.0%, 2.0% and the hydrogels were named as GCGSEs 0, GCGSEs 0.25, GCGSEs 0.5, GCGSEs 1.0 and GCGSEs 2.0. Finally, the cellulose/GSEs films (named CGSEs 0, CGSEs 0.25, CGSEs 0.5, CGSEs 1.0 and CGSEs 2.0) were fabricated after drying the hydrogels at 100 °C for 5 min using Kessel paper dryer (Lentine, Bavaria, Germany).

### 2.3. Characterization

#### 2.3.1. Wide-Angle X-ray Diffraction (WAXD) of P-OCTs, GSEs, CGSEs 0, CGSEs 0.25, CGSEs 0.5, CGSEs 1.0 and CGSEs 2.0

X-ray diffractometer D8 AD-VANCE purchased from the Bruker, Ettlingen, Germany in reflection mode with CuKa (λ = 1.5406 Å) radiation was employed to record the X-ray diffractograms (XRD) of raw materials (P-OCTs and GSEs) and the regenerated cellulose/GSEs films (CGSEs 0, CGSEs 0.25, CGSEs 0.5, CGSEs 1.0 and CGSEs 2.0), in which 40 kV, 40 mA, 20 °/min and 2θ span ranging from 5° to 60° were preset for all samples [45,48].

#### 2.3.2. Ultraviolet and Visible (UV-Vis) Spectra of the Cellulose/GSEs Films

UV 2600 Ultraviolet spectrophotometer bought from Shimadzu, Kyoto, Japan was adopted to measure the absorbance and transmittance of CGSEs 0, CGSEs 0.25, CGSEs 0.5, CGSEs 1.0 and CGSEs 2.0 films in the ultraviolet and visible region (200–800 nm). The averaged transmittance in the region of UV-A and UV-B were used to judge the ultraviolet blocking capacity as per previous references [1,45,55]:

(1) $$\mathrm{UV}-B\;\mathrm{blocking}\;\left(\%\right)=100\%-T_{\mathrm{UV}-B}$$

(2) $$\mathrm{UV}-A\;\mathrm{blocking}\;\left(\%\right)=100\%-T_{\mathrm{UV}-A}$$

in which T_UV-B_ and T_UV-A_ are the averaged T values in UV-B (280–315 nm) and UV-A (315–400 nm) regions.

#### 2.3.3. The Surface Hydrophilicity of CGSEs 0, CGSEs 0.25, CGSEs 0.5, CGSEs 1.0 and CGSEs 2.0

OCA 50 machine purchased from Dataphysics, Filderstadt, Germany was employed to record the surface hydrophilicity of CGSEs 0, CGSEs 0.25, CGSEs 0.5, CGSEs 1.0 and CGSEs 2.0. More than five positions were recorded and the averaged contact-angle values were displayed in this work [45,48].

#### 2.3.4. Fourier-Transform Infrared (FTIR) Spectra of the P-OCTs, GSEs, CGSEs 0, CGSEs 0.25, CGSEs 0.5, CGSEs 1.0 and CGSEs 2.0

ALPHA Fourier-transform infrared spectrometer bought from Bruker, Ettlingen, Germany in the attenuated total reflectance mode (4000 cm^−1^ to 700 cm^−1^) was used to investigate the structure of CGSEs 0, CGSEs 0.25, CGSEs 0.5, CGSEs 1.0, CGSEs 2.0, P-OCTs and GSEs, in which 4 cm^−1^ of the resolution and 32 scans were preset. Germanium (Ge) crystal should be washed thoroughly by ethanol between two tests. The OPUS software version 7.5 (Bruker, Ettlingen, Germany) was used to deal with the data [45,48].

#### 2.3.5. Micro-Morphologies of the Cellulose/GSEs Films

COXEM EM-30 Plus SEM microscope (Coxem, Daejeon, Korea) bought from Korea was used to create the scanning electron micrographs (SEM) of the cellulose/GSEs films and a thin layer of gold was sprayed on surface of all samples [45,48].

#### 2.3.6. Thermogravimetric Analysis (TGA) of the P-OCTs, GSEs, CGSEs 0, CGSEs 0.25, CGSEs 0.5, CGSEs 1.0 and CGSEs 2.0

TA Q50 thermogravimetric analyzer bought from United States (TA Instrument, New Castle, DE, USA) was used to record the thermal decomposition property of P-OCTs, GSEs and the regenerated cellulose/GSEs films (CGSEs 0, CGSEs 0.25, CGSEs 0.5, CGSEs 1.0 and CGSEs 2.0) in nitrogen atmosphere from 50–800 °C. Around 5 mg of samples were put into the ceramic crucible and 15 °C/min of heating rate was set for all samples [45,48].

#### 2.3.7. Mechanical Tests of CGSEs 0, CGSEs 0.25, CGSEs 0.5, CGSEs 1.0 and CGSEs 2.0

Stable Micro System TA.XT Plus C (Stable Micro Systems Ltd., Godalming, UK) texture analyzer bought from UK was employed to measure the mechanical property of CGSEs 0, CGSEs 0.25, CGSEs 0.5, CGSEs 1.0 and CGSEs 2.0 films, in which a 5 kN load cell, 20 mm of the gauge length and 4.8 mm min^−1^ of drawing speed were adopted. All cellulose/GSEs films were cut into at least six strips with approximately 10 mm in width and 60 mm in length [45,48].

#### 2.3.8. Barrier Properties of CGSEs 0, CGSEs 0.25, CGSEs 0.5, CGSEs 1.0 and CGSEs 2.0

Water-vapor transmission rate (WVTR) Tester (W3/060, Labthink, Jinan, China) was used to measure the barrier properties of CGSEs 0, CGSEs 0.25, CGSEs 0.5, CGSEs 1.0 and CGSEs 2.0 films by reference of GB1037 or ASTM E96 standard [1,55,56]. The cellulose/GSEs films were put into the a cabinet for at least 48 h to equilibrate before the water vapor transmission rate test, where the humidity and temperature were 50% RH and 25 °C. All samples were cut into 9 cm diameter circles and sealed in the glass cup. Then, all glass cups with samples were put into the box of permeability meter at 50% RH and 25 °C, where desiccating agent was molecular sieve. Water vapor passed through the cellulose/GSEs films to the dry side during the test and the weight of the cups sealed with the sample films were recorded periodically. Finally, water vapor transmission rate WVTR (g/(m^2^day)) of all samples was obtained by referring to the following Equation (3).

(3) $$\mathrm{WVTR}=\frac{\Delta \omega }{A\Delta t}$$

where ∆t, A and ∆ω are a definite time, a film area (A) and moisture weight gain, respectively.

#### 2.3.9. Statistical Analysis

Statistical analysis was performed employing the IBM SPSS Statistics v 19.0.0 (IBM, Armonk, NY, USA) and differences were considered to be significant at a level of *p* < 0.05.

## 3. Results

### 3.1. Transparency of Cellulose/GSEs Hydrogels and Films

**Figure 2:**
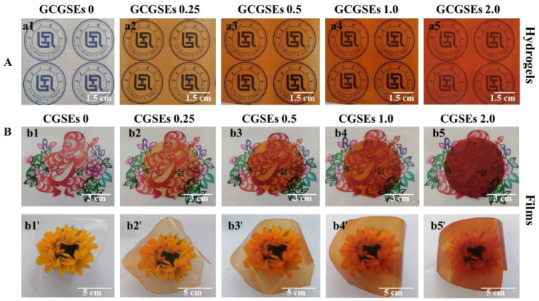
(**A**,**B**) Digital photographs of cellulose/GSEs composite hydrogels (**a1**) GCGSEs 0, (**a2**) GCGSEs 0.25, (**a3**) GCGSEs 0.5, (**a4**) GCGSEs 1.0, (**a5**) GCGSEs 2.0) and films (**b1**,**b1’**) CGSEs 0, (**b2**,**b2’**) CGSEs 0.25, (**b3**,**b3’**) CGSEs 0.5, (**b4**,**b4’**) CGSEs 1.0, (**b5**,**b5’**) CGSEs 2.0) against different background.

The digital photographs of cellulose/GSEs composite hydrogels and films against different backgrounds and their simulated continuous production method in large scale is demonstrated in Figure 2. It is known that raw regenerated cellulose materials, including hydrogels, films and fibers, are transparent and colorless in the visible light region. As displayed in Figure 2(a1,b1,b1′), GCGSEs 0 and CGSEs 0 made from the pretreated old cotton textiles (P-OCTs) are colorless and transparent, meaning that P-OCTs are decolored completely and the quality of P-OCTs is high. Different from the pure cellulose materials GCGSEs 0 and CGSEs 0, the cellulose/GSEs composite hydrogels and films show an obvious brown color after immersion in the GSEs/glycerol/water coagulation. Furthermore, the color grows darker and darker with increasing GSEs content in the last coagulation bath, suggesting that more of the content of GSEs migrated from coagulation to the cellulose/GSEs composite materials. Meanwhile, it can be concluded that GSEs are distributed evenly in the composite hydrogels and films. It is worth noting that the cellulose/GSEs composite gels and films still display high transparency and the background of the paper-cutting monkey is still clear to see, although the content of GSEs in the last coagulation is as high as 2.0% (Figure 2(b5)), indicating that adding GSEs can impart a homogenous brown color to the composite materials without damaging their transparency significantly. Additionally, cellulose/GSEs films exhibit enough flexibility, which is necessary for packaging materials (Figure 2(b1’–b5’)). In this work, cellulose/GSEs composite hydrogels and films with 20 cm in length and 10 cm in width were prepared. In fact, the cellulose films regenerated using the ionic liquid method were successfully fabricated by the ICCAS-Henglian Biobased Materials Co., Ltd. (Weifang City, China) Therefore, a simulated continuous production line can be designed to produce the cellulose/GSEs composite film at a large scale, where a portable coagulation bath unit can be merged with the existed continuous production line. In conclusion, a relatively green, feasible and cost-effective method is put forward to produce the transparent cellulose/GSEs composite hydrogels and films.

### 3.2. Ultraviolet and Visible (UV-Vis) Spectra of Cellulose/GSEs Composite Films

**Figure 3:**
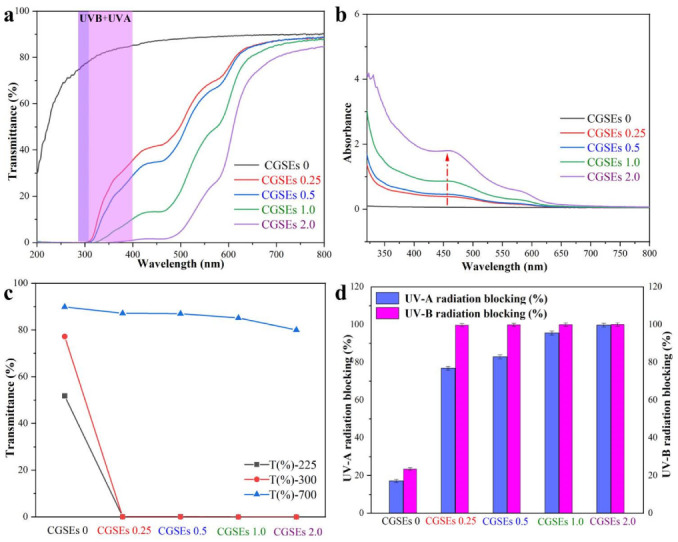
Transmittance (**a**) and absorbance (**b**) of cellulose/GSEs composite films; (**c**) T(%)-700, T(%)-300 and T(%)-225 curves of cellulose/GSEs composite films; (**d**) UV-B and UV-A blocking values of cellulose/GSEs composite films.

Generally, transparency is important for packaging materials. To quantitatively describe the transparency of CGSEs 0, CGSEs 0.25, CGSEs 0.5, CGSEs 1.0 and CGSEs 2.0 films, their absorbance and transmittance are output (Figure 3a,b). Meanwhile, T(%)-700, T(%)-300, T(%)-225, UV-B and UV-A blocking values are also displayed in Figure 3c and d to assess their anti-ultraviolet capacity. It can be seen that the pure cellulose film without any GSEs (CGSE0) display high transmittance and shows no obvious absorption peak during 200–800 nm. Specially, the transmittance of CGSE0 is above 85.0% in the visible light region and possesses no obvious anti-ultraviolet capacity, which is similar to that of traditional cellophane (Figure 3a,b). It is reported that grape seed extracts have anti-oxidation and other advantages, Nevertheless, the anti-ultraviolet capacity of grape seed extracts is rarely shown in the previous investigations. Thus, the anti-ultraviolet cellulose/GSEs films were fabricated successfully by the incorporation of GSEs in this work, because GSEs exhibit intensive absorption peaks below 600 nm. It can be noticed that the transmittance of all the cellulose/GSEs films is nearly 0% in the region of 200–300 nm, when the content of GSEs in the coagulation bath is more than 0.25%. Fortunately, the anti-ultraviolet capacity of the cellulose/GSEs films are enhanced by increasing the GSEs content and the transmittance of the cellulose/GSEs composite films is below 1.0% in the whole ultraviolet region (200–400 nm) when the content of GSEs is more than 2.0% in the coagulation bath, indicating that the anti-ultraviolet capacity of grape seed extracts is stronger than that of the tea polyphenols reported in our previous work [1].

Additionally, the high anti-ultraviolet capacity and good transparency of the cellulose/GSEs films can also be proved by the T(%)-700, T(%)-300 and T(%)-225 values (Figure 3c). It can be concluded than the values of T(%)-300 and T(%)-225 decreased dramatically, while the T(%)-700 values changed little. Meanwhile, the UV-B and UV-A blocking values of CGSEs 0, CGSEs 0.25, CGSEs 0.5, CGSEs 1.0 and CGSEs 2.0 films were also calculated, according to the previous works, in Figure 3d [1,55]. It can be seen that the UV-B and UV-A blocking values of CGSE0, the pure cellulose film, are about 23% and 17%, while those of CGSE 2.0, the composite film, are as high as nearly 100% for both UV-B and UV-A, proving that cellulose/GSEs films possess good UV-blocking performance due to the addition of GSEs. Briefly speaking, the cellulose/GSEs films show super transparence and anti-ultraviolet capacity, which are good for packaging applications.

### 3.3. Mechanical and Hydrophilicity Performances of CGSEs 0, CGSEs 0.25, CGSEs 0.5, CGSEs 1.0 and CGSEs 2.0 Films

**Figure 4:**
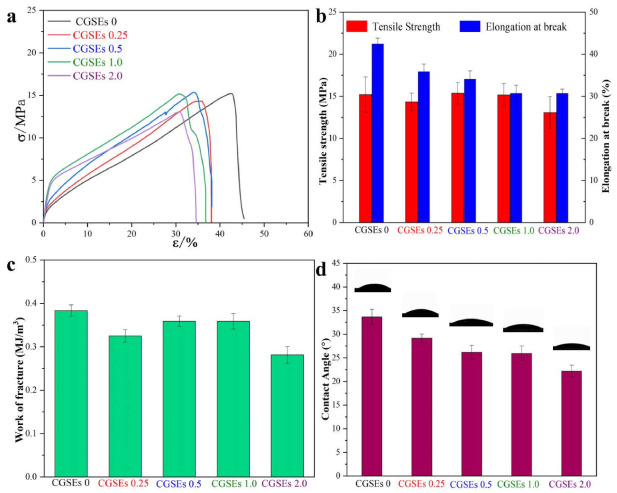
Stress–strain curves (**a**), tensile strength and elongation at break (**b**), work of fracture (**c**) and surface wettability (**d**) of the cellulose/GSEs composite films.

The most important property for the application of the polymer films is their mechanical property. As shown in Figure 4a–c, the mechanical properties of CGSEs 0, CGSEs 0.25, CGSEs 0.5, CGSEs 1.0 and CGSEs 2.0 films were evaluated by uniaxial tension tests. It can be concluded that the CGSEs 0 film produced from the raw pretreated old cotton textiles still shows good mechanical property though OCTs had been worn for more than 7 years, because the degree of polymerization (DP) of the cellulose in p-OCTs was approximately 300 and it changed little after regeneration from AmimCl [34,35]. In addition, the tensile strength of the CGSEs 0 film is around 15.2 MPa and it vibrates weakly after the addition of GSEs, where the tensile strengths of the CGSEs 0.25, CGSEs 0.5, CGSEs 1.0 and CGSEs 2.0 are around 14.3 MPa, 15.4 MPa, 15.2 MPa and 13.1 MPa, respectively. Generally, the elongation at the break of cellulose films regenerated from the ionic liquids or other solvents is usually below 5% without any additions [34,37]. Nevertheless, the elongation at the break can be improved by a plasticizer, such as glycerol. Thus, the elongations at the break of the films CGSEs 0, CGSEs 0.25, CGSEs 0.5, CGSEs 1.0 and CGSEs 2.0 are about 42.5%, 35.9%, 34.1%, 30.7%, and 30.7%, respectively, higher than those of most regenerated films without any plasticizers. It is worth mentioning that the elongation at the break of polymer materials can be obviously improved by increasing the content of the plasticizer, but their tensile strength will be decreased, conversely. In addition, the work of a fracture can be used to assess the toughness of the polymer materials [35]. As displayed in Figure 4c, the toughness of the cellulose/GSEs composite films CGSEs 0, CGSEs 0.25, CGSEs 0.5, CGSEs 1.0 and CGSEs 2.0 is about 0.383 MJ/m^3^, 0.325 MJ/m^3^, 0.359 MJ/m^3^, 0.359 MJ/m^3^ and 0.281 MJ/m^3^, respectively, indicating their good toughness. Briefly speaking, cellulose/GSEs films show the relatively high mechanical properties required to be adopted as wrapping and packaging materials, but the incorporation of GSEs exhibits no significant impact on their mechanical property.

The surface wettability of CGSEs 0, CGSEs 0.25, CGSEs 0.5, CGSEs 1.0 and CGSEs 2.0 films was also evaluated by the water contact angles, as demonstrated in Figure 4d. It can be noticed that CGSEs 0, the pure cellulose film fabricated from the raw P-OCTs, displays good hydrophilicity and its water contact angle is about 33.7°. Meanwhile, the water contact angles of the cellulose/GSEs composite films decreased with increasing contents of GSEs, from 0.25% to 2.0%, which may be ascribed to the high hydrophilicity of the GSEs. Particularly, the water contact angles of CGSEs 0.25, CGSEs 0.5, CGSEs 1.0 and CGSEs 2.0 are around 29.2°, 26.2°, 26.0°and 22.2°, respectively. As a result, the cellulose/GSEs composite films possess good hydrophilicity.

### 3.4. TGA, FTIR and XRD of Cellulose/GSEs Hybrid Films

**Figure 5:**
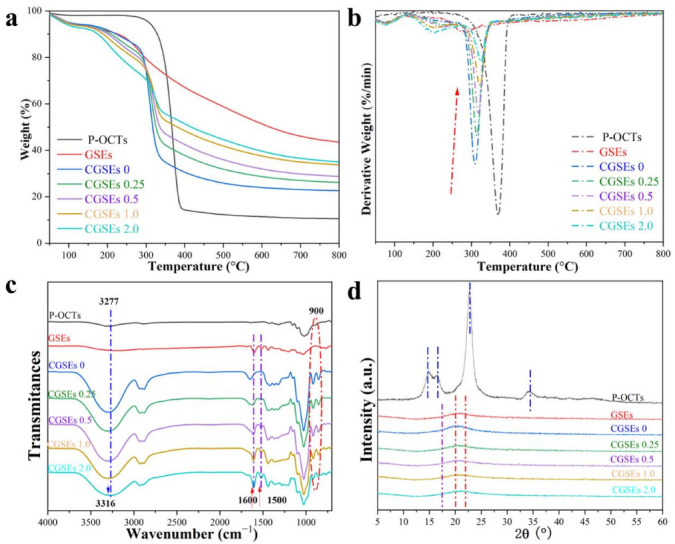
Thermogravimetric curves (**a**), derivative thermogravimetric curves (**b**), Fourier transform infrared spectroscopy (**c**) and X-ray diffractograms (**d**) of the cellulose/GSEs films and their raw material.

Figure 5a,b demonstrates the thermogravimetric (TG) and derivative thermogravimetric data (DTG) of CGSEs 0, CGSEs 0.25, CGSEs 0.5, CGSEs 1.0, CGSEs 2.0 films and their raw material P-OCTs and GSEs. In general, the mass loss at the temperature lower than 135 °C of all samples is attributed to the loss of moisture [35]. Meanwhile, it can be concluded that the P-OCTs start to degrade at about 250 °C and the temperature of maximum weight loss rates (T_max_) is at approximately 370 °C, displaying the highest thermal stability. By contrast, the cellulose/GSEs composite films decompose at the temperature higher than 135 °C and the T_max_ of the them are ranging from 300°C to 330 °C, meaning the decreased thermal stability of CGSEs 0, CGSEs 0.25, CGSEs 0.5, CGSEs 1.0, CGSEs 2.0 films, which is ascribed to transformation of cellulose structure after the dissolution and regeneration process. However, the thermal stability of the cellulose/GSEs composite films is better than that of the traditional polyethylene (PE) film and the PE starts to melt when the temperature is above 100 °C, indicating the superiority of the cellulose/GSEs composite films when employed in the wrapping and packaging fields. Meanwhile, it can be seen that the T_max_ of GSEs is about 290 °C and the T_max_ of the cellulose/GSEs composite films is increased obviously by increasing the content of GSEs in the composite films. For example, the T_max_ of CGSEs 0 is around 309 °C and it is increased to 314 °C when the content of GSEs in the last coagulation is 0.25% and, finally, it is increased to as high as 325 °C when the content of GSEs is 2.0%, about 16 °C higher than that of CGSEs 0. Additionally, the main component of GSEs is anthocyanin and many benzene rings in its structures; thus, the residual mass of the GSEs is 43.62% at 797 °C, higher than those of P-OCTs (10.55%) and the cellulose/GSEs composite films (22–33%). Moreover, the residual mass of the cellulose/GSEs films is increased obviously with raising contents of GSEs. In short, the results mean that that incorporation of GSEs enhance the thermal stability of the cellulose/GSEs composite films, which may be ascribed to the intensive hydrogen bonding interactions among their molecules.

To obtain more information about the components and structures of the cellulose/GSEs composite films and their raw materials, their FTIR curves were recorded and are shown in Figure 5c. It can be concluded that the regenerated CGSEs 0 film displays a similar spectrum to that of the raw material P-OCTs, meaning that the AmimCl is a non-derivative solvent for cellulose [34,35,54]. Nevertheless, the spectra of CGSEs 0, CGSEs 0.25, CGSEs 0.5, CGSEs 1.0, and CGSEs 2.0 films are obviously different from those of P-OCTs and CGSEs 0. Obviously, the cellulose/GSEs composite films show two distinct peaks at around 1500 cm^−1^ and 1600 cm^−1^, which are feature peaks of aromatic rings in GSEs [1,57]. Thus, the raw GSEs possess prominent peaks at above wavenumber. Meanwhile, the intensity of these two peaks is enhanced with the increasing content of GSEs, which is in conformity with established results. Moreover, there are no new peaks in the spectra of the cellulose/GSEs composite films, suggesting that cellulose and GSEs are physically mixed together. Additionally, the blue shift in the O-H stretching peaks ranging from 3200 cm^−1^ to 3700 cm^−1^ is the indication of the changes in crystalline structure and hydrogen bonding [1,34]. It can be noticed that the maximum absorption peaks of O-H stretching is at 3277 cm^−1^ for the P-OCTs, while this peak moves to 3316 cm^−1^ for the CGSEs 2.0, meaning that the structure of cellulose changes a lot after the dissolution and regeneration process. Moreover, the bands of the regenerated films (CGSEs 0, CGSEs 0.25, CGSEs 0.5, CGSEs 1.0 and CGSEs 2.0) at approximately 900 cm^−1^ are different from that of the raw P-OCTs. All these prove that there is a phase transformation of cellulose after the dissolution and regeneration process.

XRD was conducted to analyze the phase structures of CGSEs 0, CGSEs 0.25, CGSEs 0.5, CGSEs 1.0, CGSEs 2.0 films and their raw materials (GSEs and P-OCTs). The peaks at 16.7° (110), 22.8° (200), 34.6° (004) and 14.8° (1–10) are assigned to the I phase of cellulose and the P-OCTs show obvious peaks at the above positions, indicating that P-OCTs are cellulose I phase, which is in correspondence with previous works [35]. Meanwhile, the GSEs show no significant diffraction peaks due to their amorphous components. By contrast, the XRD curves of film CGSEs 0 and the cellulose/GSEs composite films are different from that of raw P-OCTs, suggesting a dramatic change in phase structures. Generally, the cellulose changes from phase I to II after the dissolution and regeneration process and the degree of crystallinity will also decrease a lot during this process [37,43]. Thus, the intensity of P-OCTs is higher than those of the CGSEs 0, CGSEs 0.25, CGSEs 0.5, CGSEs 1.0, and CGSEs 2.0 films. Moreover, CGSEs 0 and other cellulose/GSEs composite films exhibit a broad peak only at about 2θ = 20.0°, which is attributed to the merged peaks of amorphous cellulose and minor cellulose II phase [43]. In addition, CGSEs 0.25, CGSEs 0.5, CGSEs 1.0, and CGSEs 2.0 films display similar diffractograms, indicating that the incorporation of GSEs show no significant impact on the crystallization of the cellulose.

### 3.5. Barrier Property of the Cellulose/GSEs Film

**Figure 6:**
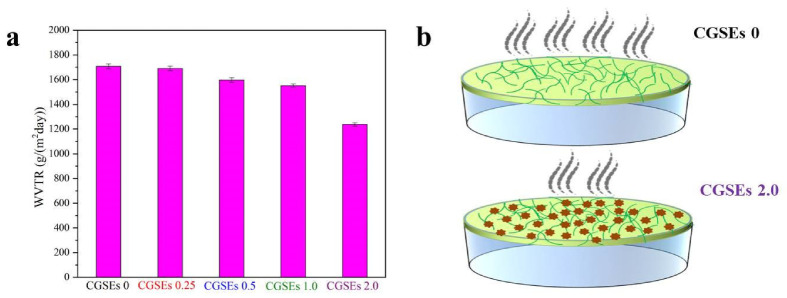
(**a**) Water vapor transmission rate of the cellulose/GSEs film; (**b**) the schematic comparison of the CGSEs 0 and CGSEs 2.0 film.

As displayed in Figure 6a, water vapor transmission rate (WVTR) is used to evaluate the water vapor barrier properties of CGSEs 0, CGSEs 0.25, CGSEs 0.5, CGSEs 1.0, and CGSEs 2.0 films, and the GB1037 or ASTM E96 standard method were used as the reference [55,56,58]. The WVTR value of CGSEs 0 is 1708 g/(m^2^day) and it is reduced by raising the content of GSEs, where WVTR values of CGSEs 0.25, CGSEs 0.5, CGSEs 1.0 and CGSEs 2.0 are 1691 g/(m^2^day), 1597 g/(m^2^day), 1552 g/(m^2^day) and 1237 g/(m^2^day), respectively. By contrast, the WVTR value of CGSEs 0 is about 27.5% higher than that of film CGSEs 2.0, meaning that the existence of GSEs obviously impacts the water vapor barrier performance of the cellulose-based films, which is also consistent with our previous work [1]. The reason for this may be that GSEs molecules in the cellulose/GSEs composite films block the water-vapor-molecule diffusion by occupying the free space for water vapor to migrate and the schematic comparison of the CGSEs 0 and CGSEs 2.0 film is shown in Figure 6b, resulting in the decrease of the WVTR of CGSEs 2.0 film [55]. Additionally, it is known that petrochemical-based plastics, including those used widely in our daily life, show lower water-vapor permeability than those of cellulose/GSEs composite films [1,55], meaning that the cellulose/GSEs composite films are more fit for fresh packaging applications.

### 3.6. Micro-Morphologies of the Cellulose/GSEs Films

**Figure 7:**
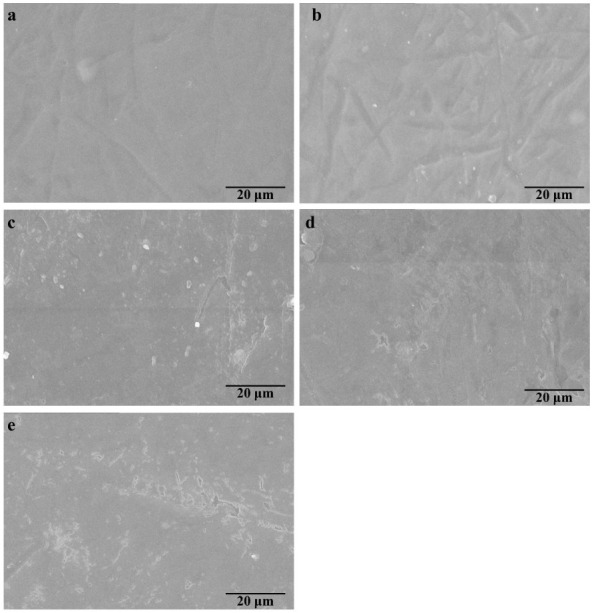
Micro-morphologies of the cellulose/GSEs films: the surface (**a**–**e**) of CGSEs 0, CGSEs 0.25, CGSEs 0.5, CGSEs 1.0 and CGSEs 2.0.

Scanning electron micrographs (SEM) were employed to investigate the surficial micro-images of CGSEs 0, CGSEs 0.25, CGSEs 0.5, CGSEs 1.0, and CGSEs 2.0 films. As displayed in Figure 7, all films show relatively homogeneous surfaces, suggesting that GSEs are distributed relatively uniformly in the cellulose matrix, which is important to their mechanical property. Nevertheless, the cellulose/GSEs films are adhered to by some small particles during the preparation process. Meanwhile, their surfaces become rougher with increasing the content of grape seed extracts, indicating that grape seed extracts may have an effect on the morphology of the cellulose/GSEs films in this work.

## 4. Conclusions

Using a relatively simple, green and cost-effective approach, renewable biodegradable and transparent all-biomass cellulose/grape seed extracts (GSEs) composite films with high anti-ultraviolet performance were manufactured successfully from pretreated old cotton textiles (P-OCTs). The structure and property of cellulose/GSEs films are homogenous, which may be attributed to the intensive hydrogen-bond interactions among their molecule. Moreover, there are no chemical interactions in the preparation of cellulose/GSEs films and the dissolution of cellulose and GSEs in ionic liquid are physical processes. It was proved that cellulose/GSEs films possess super ultraviolet shielding performance without sacrificing their transparency, and both UV-B and UV-A blocking values of the cellulose/GSEs composite materials can reach as high as nearly 100%, when the content of GSEs in the last coagulation is 2.0%, higher than those of the pure cellulose (CGSEs 0) film (UVA: 17% and UVB: 23%), indicating the good UV-blocking capacity of GSEs. Meanwhile, the mechanical property of the cellulose/GSEs film can be adjusted by the addition of a plasticizer. The elongation at the break of polymer materials is obviously improved by increasing the content of the plasticizer glycerol, but their tensile strength will be decreased, conversely. Furthermore, the thermal stability of the cellulose/GSEs film is improved with raising contents of GSEs, the temperature of the maximum weight loss rates (T_max_) of pure CGSEs 0 film is around 309 °C and it, finally, increased to 325 °C, suggesting that GSEs enhance the thermal stability of the cellulose/GSEs composite films due to the intensive hydrogen bonding interactions among their molecules. In addition, the residual mass of cellulose/GSEs films also increased obviously with the increasing addition of GSEs, because of copious benzene rings in the skeleton structure of GSEs. Additionally, the addition of GSEs decreases the water contact angles and water vapor transmission rate of the cellulose/GSEs films. Nevertheless, the cellulose/GSEs films show higher water vapor transmission rate and thermal stability than those of most common plastics, which are important for them to be used as packaging materials. Therefore, this kind of transparent and anti-ultraviolet all-biomass cellulose/grape seed extracts composite film can be used as a potential material in packaging fields.

## Data Availability

Data is available from the corresponding author on reasonable request.
